# Supplementary material for: Production of [11C]Carbon Labelled Flumazenil and L-Deprenyl Using the iMiDEV™ Automated Microfluidic Radiosynthesizer
Source: Molecules. 2022 Dec 13;27(24):8843. doi: 10.3390/molecules27248843 (PMC9788284; doi:10.3390/molecules27248843)
Supplement: Supplementary file 1 [file molecules-27-08843-s001.zip › 1212_molecules-1985588-supplementary.pdf]

Supplementary Materials

Production of [11C]Carbon Labeled Flumazenil and *L*-Deprenyl Using the iMiDEV™ Automated Microfluidic Radiosynthesizer

Hemantha Mallapura[1,*], Laurent Tanguy[2], Bengt Långström[3], Ludovic Le Meunier[2], Christer Halldin[1], and Sangram Nag[1]

[1] Department of Clinical Neuroscience, Center for Psychiatry Research, Karolinska Institutet and Stockholm County Council, Stockholm 17176, Sweden
[2] PMB Alcen, Route des Michels CD56, F-13790 Peynier, France
[3] Department of Chemistry, Uppsala University, Sweden
* Correspondence: hemantha.mallapura@ki.se; Tel.: +46-(0)-769084212

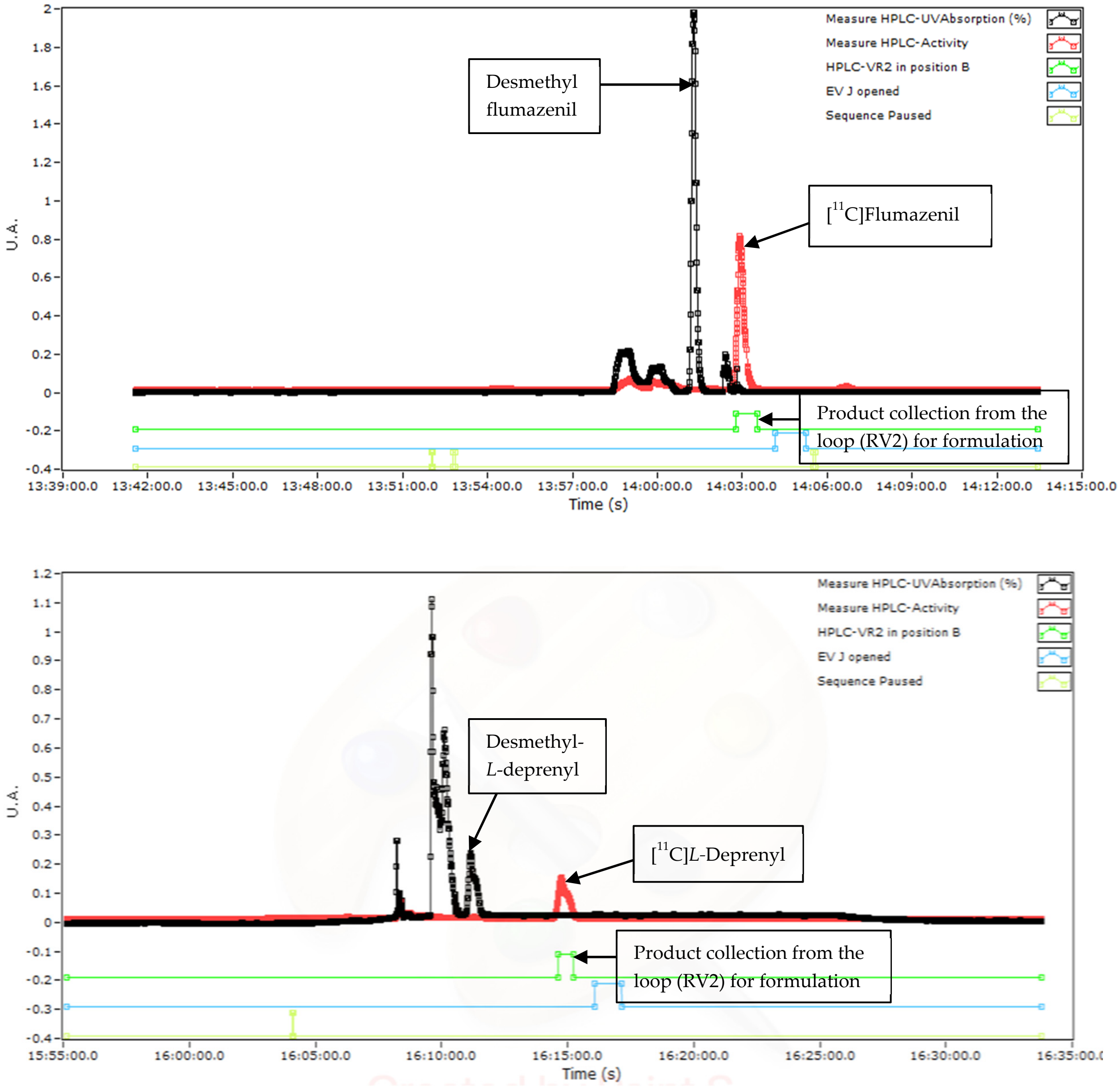

Figure S1. Semi preparative HPLC chromatograms of [11C]flumazenil and [11C]*L*-deprenyl.

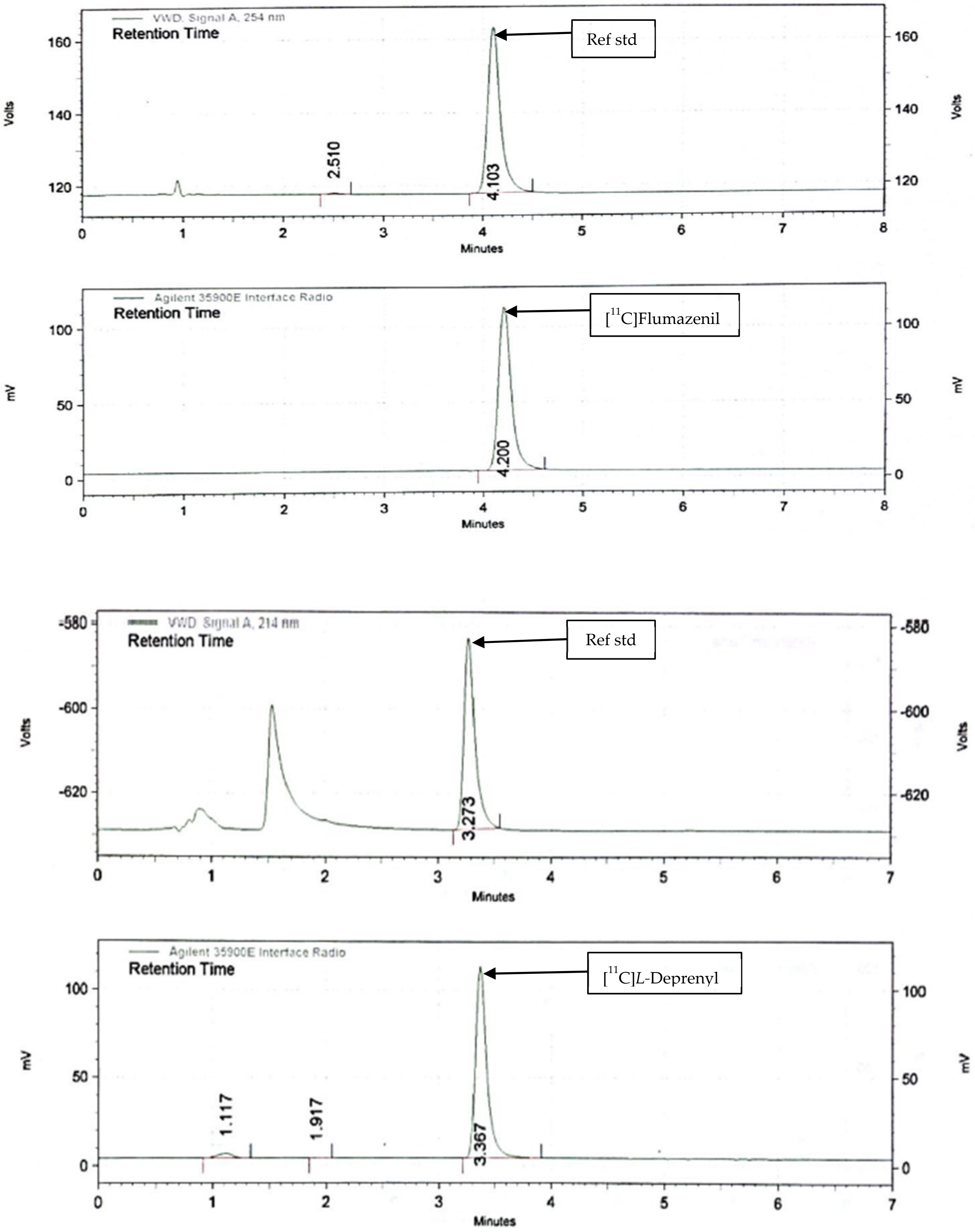

Figure S2. Analytical HPLC data of [^{11}C]flumazenil and [^{11}C]*L*-deprenyl.

Table S1. Methylating agents, solvents and bases used for [^{11}C]flumazenil synthesis.

<table>
<tr><td colspan="4">[^{11}C]Flumazenil synthesis</td></tr>
<tr><td>Methylating agent</td><td>Solvent</td><td>Base</td><td>RCC (%)</td></tr>
<tr><td rowspan="4">[^{11}C]CH_3OTf</td><td>Acetone/DEK</td><td rowspan="8">aq. NaOH</td><td rowspan="8">0-20%</td></tr>
<tr><td>Acetone</td></tr>
<tr><td>DMF</td></tr>
<tr><td>DMSO</td></tr>
<tr><td rowspan="4">[^{11}C]CH_3I</td><td>MeOH and ACN</td></tr>
<tr><td>DMSO</td></tr>
<tr><td>DMF</td></tr>
<tr><td>Acetonitrile</td></tr>
<tr><td rowspan="2">[^{11}C]CH_3I</td><td>DMSO</td><td rowspan="2">KOH powder</td><td>70%</td></tr>
<tr><td>1:1 mixture of DMSO/DMF</td><td>65-85%</td></tr>
</table>

DEK- Diethyl ketone

DMF - Dimethylformamide

DMSO – Dimethyl sulfoxide

RCC-Radiochemical conversion

Table S2. Methylating agents, solvents and bases used for the [^{11}C]*L*-deprenyl synthesis.

[^{11}C]*L*-Deprenyl synthesis			
Methylating agent	Solvent	Base	RCC (%)
[^{11}C]CH_3OTf	MeOH and ACN	PMP	> 65%
	Diethyl ketone	aq. NaOH	None
[^{11}C]CH_3I	1:1 mixture of DMSO/DMF	KOH powder	None
		PMP	
	DMF	Cs_2CO_3	
		NaH	
		TBAH	

Cs_2CO_3 – Cesium carbonate

NaH - Sodium Hydride

TBAH- Tetrabutylammonium hydroxide

PMP- Pentamethylpiperidine

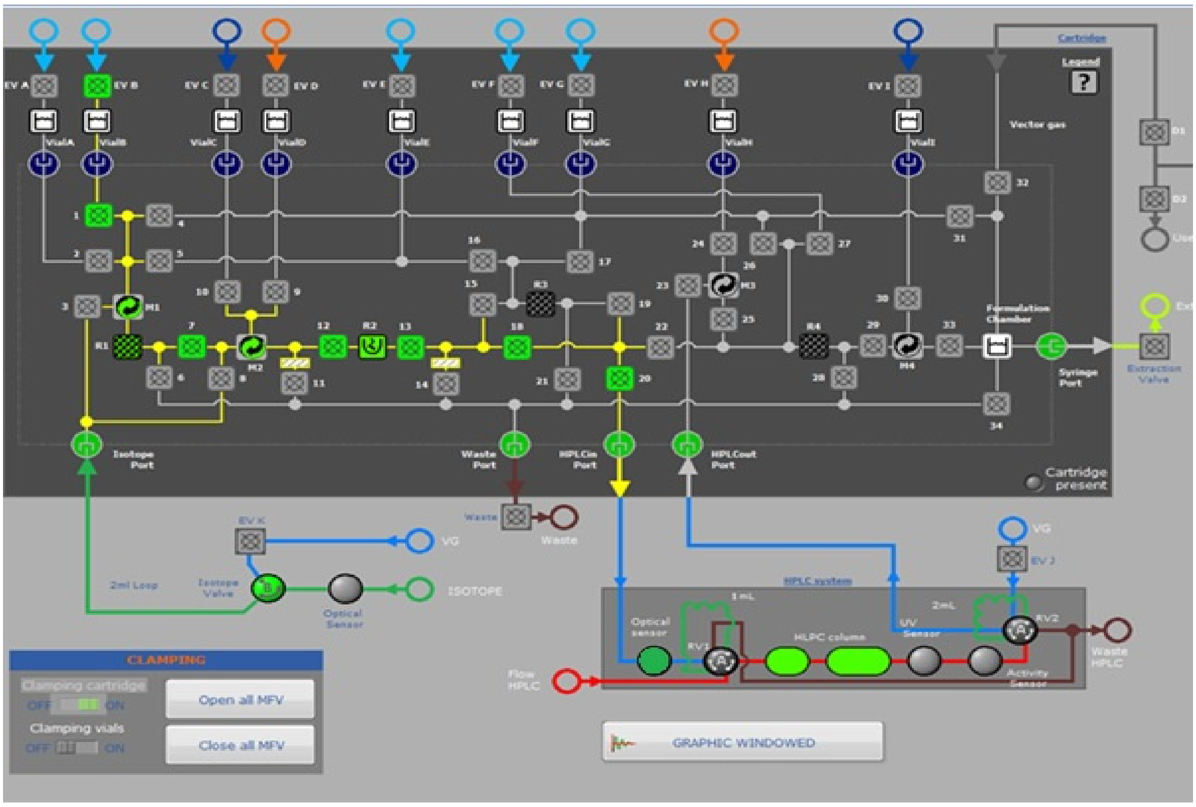

Figure S3. Semi-preparative HPLC injection from the vial B.

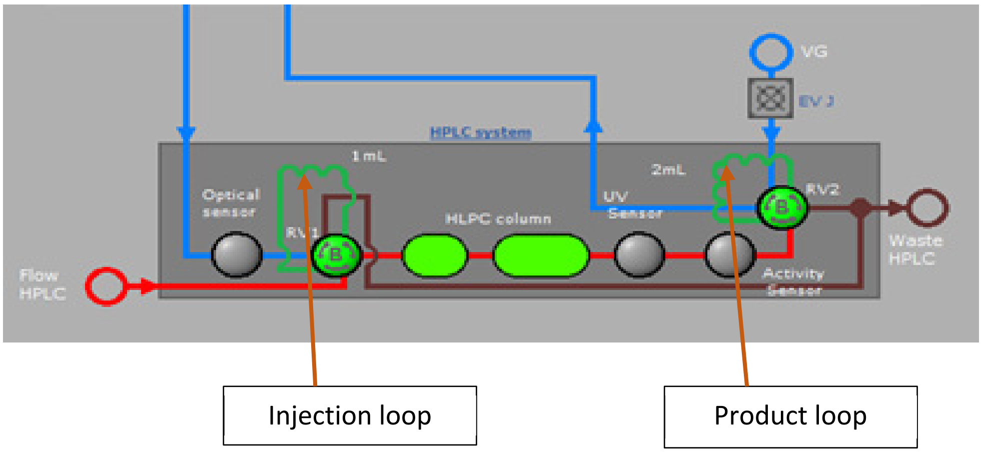

Figure S4. Semi-preparative HPLC purification (product collection) from the RV2 loop (position B).

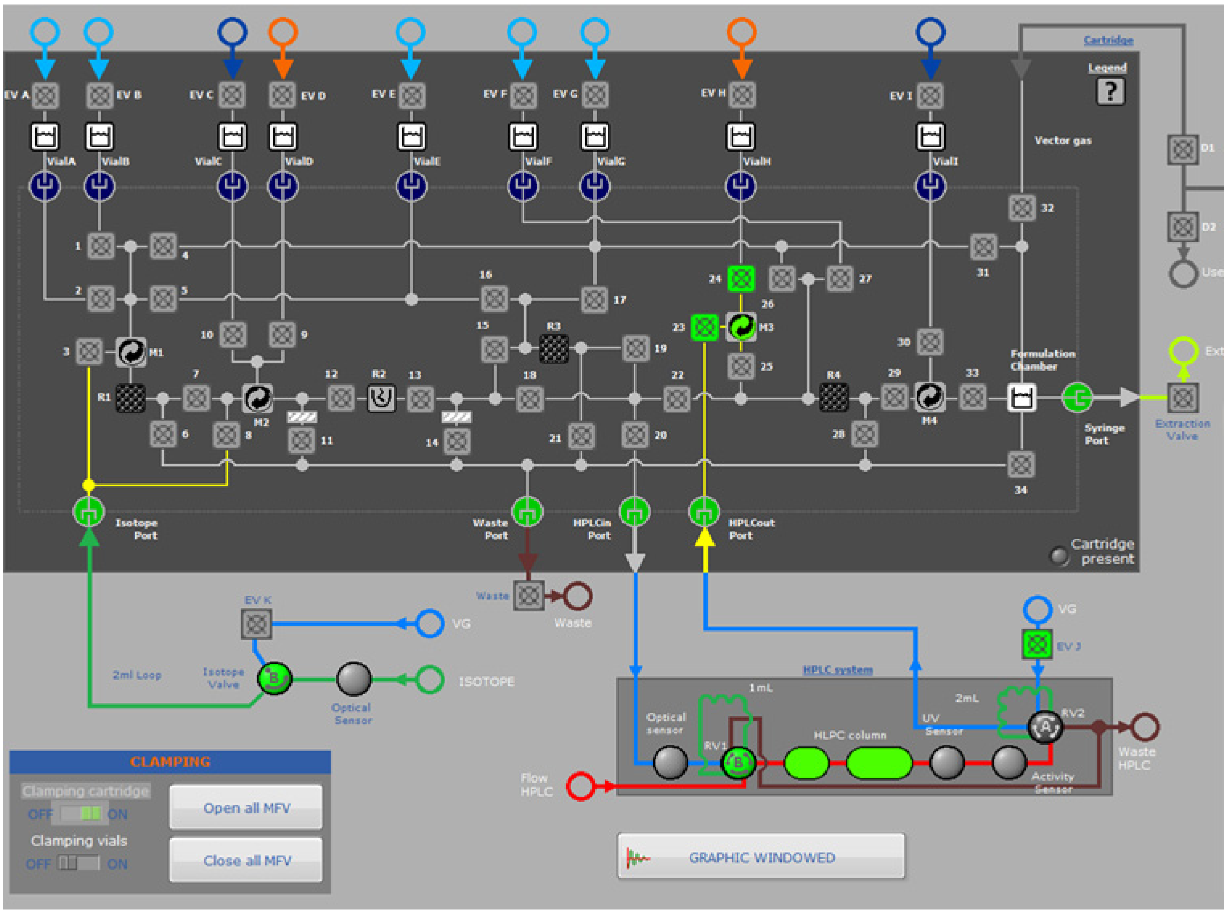

Figure S5. Pushing the product from the loop RV2 (Position A) to the vial H in the iMiDEV™ cassette.

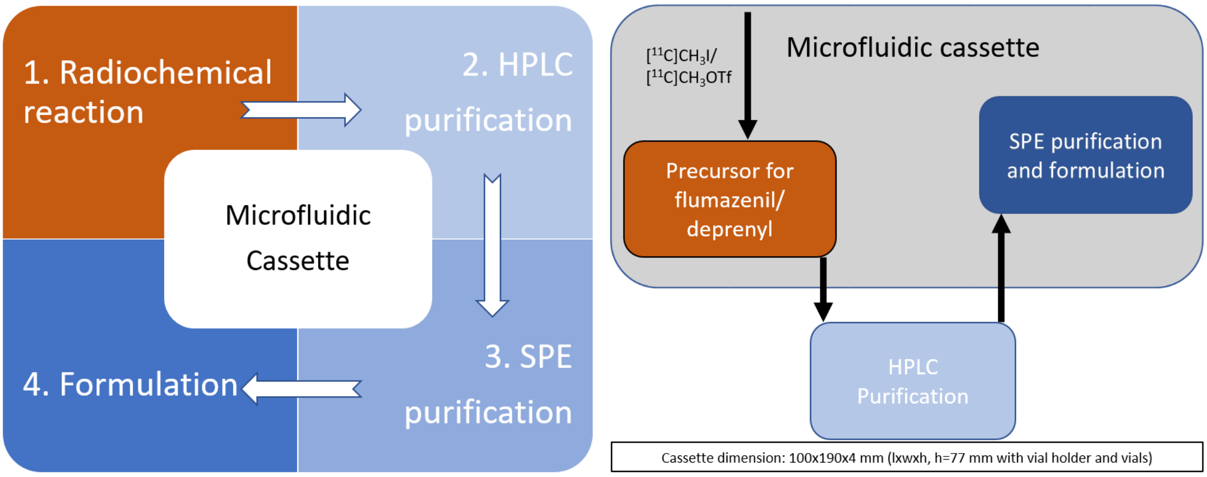

Figure S6. Flow chart of the synthesis steps performed on a microfluidic cassette.
