# Supplementary material for: Production of [11C]Carbon Labelled Flumazenil and L-Deprenyl Using the iMiDEV™ Automated Microfluidic Radiosynthesizer
Source: Molecules. 2022 Dec 13;27(24):8843. doi: 10.3390/molecules27248843 (PMC9788284; doi:10.3390/molecules27248843)
Supplement: Supplementary file 1 [file molecules-27-08843-s001.zip › iMiDEV module synthesis report.pdf]

Synthesis report of 02/11/2021 at 13:41

User	HEMANTHA
Recipe name	20211027_FMZ_R1,HPLC waste and FormIt and vial F elution_4mL_35% ACN_B_500mbar.csv
Recipe SHA1	D9CFF20ED444B9D7D676934948193191B36A01DC
Version supervision	1.0.2.8
Version API Synthèse	1.0.0.14
Version API Radiochimie	
Total time	00:31:53.760
Start hour	13:41:32.700
Stop hour	14:13:26.460

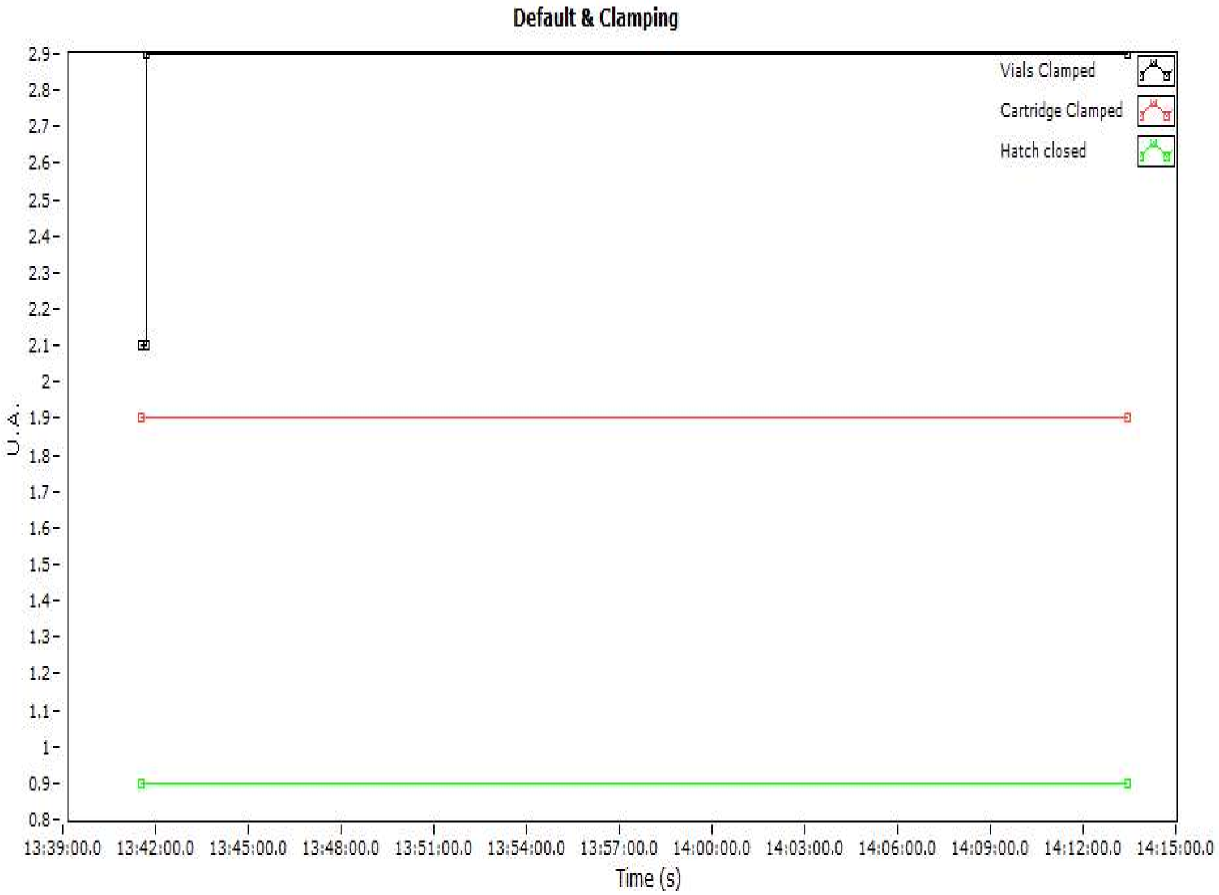

Default & Clamping

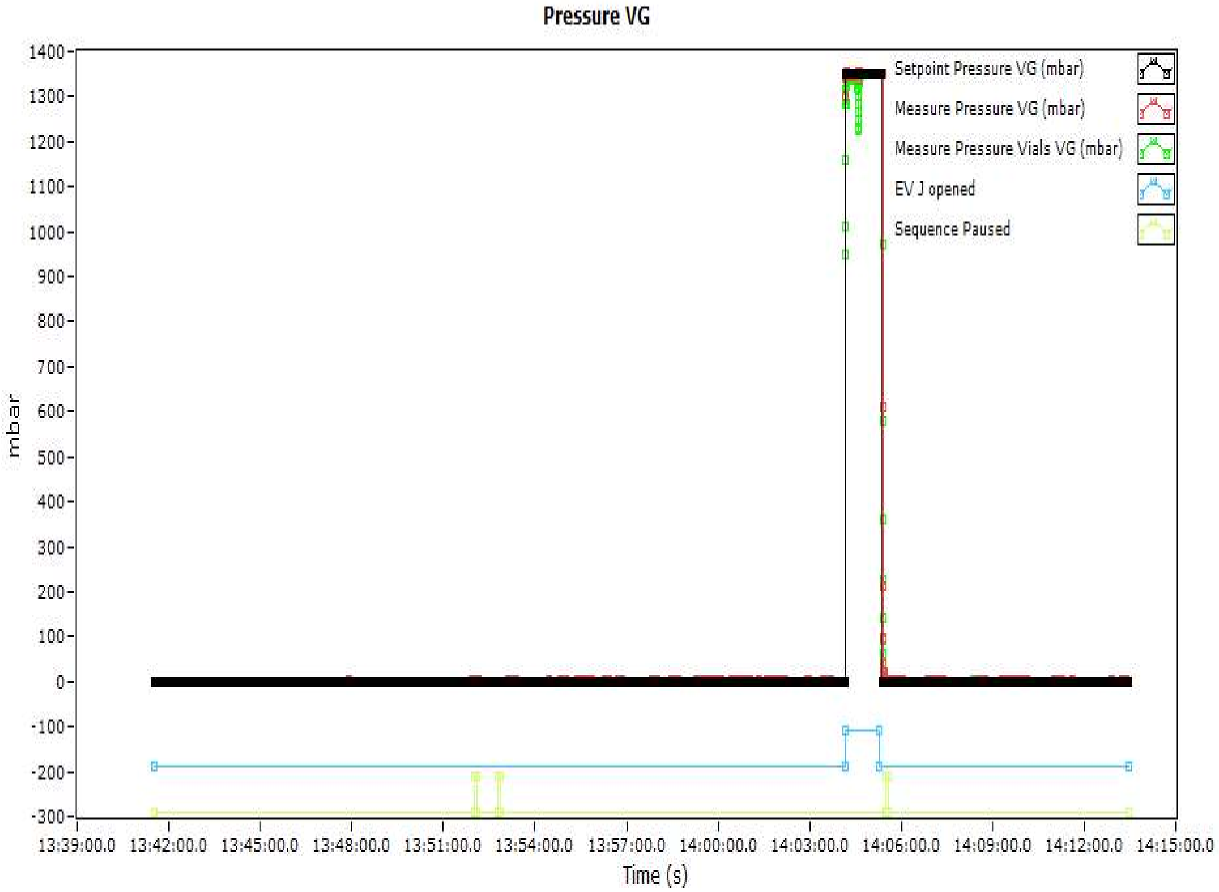

Pressure VG

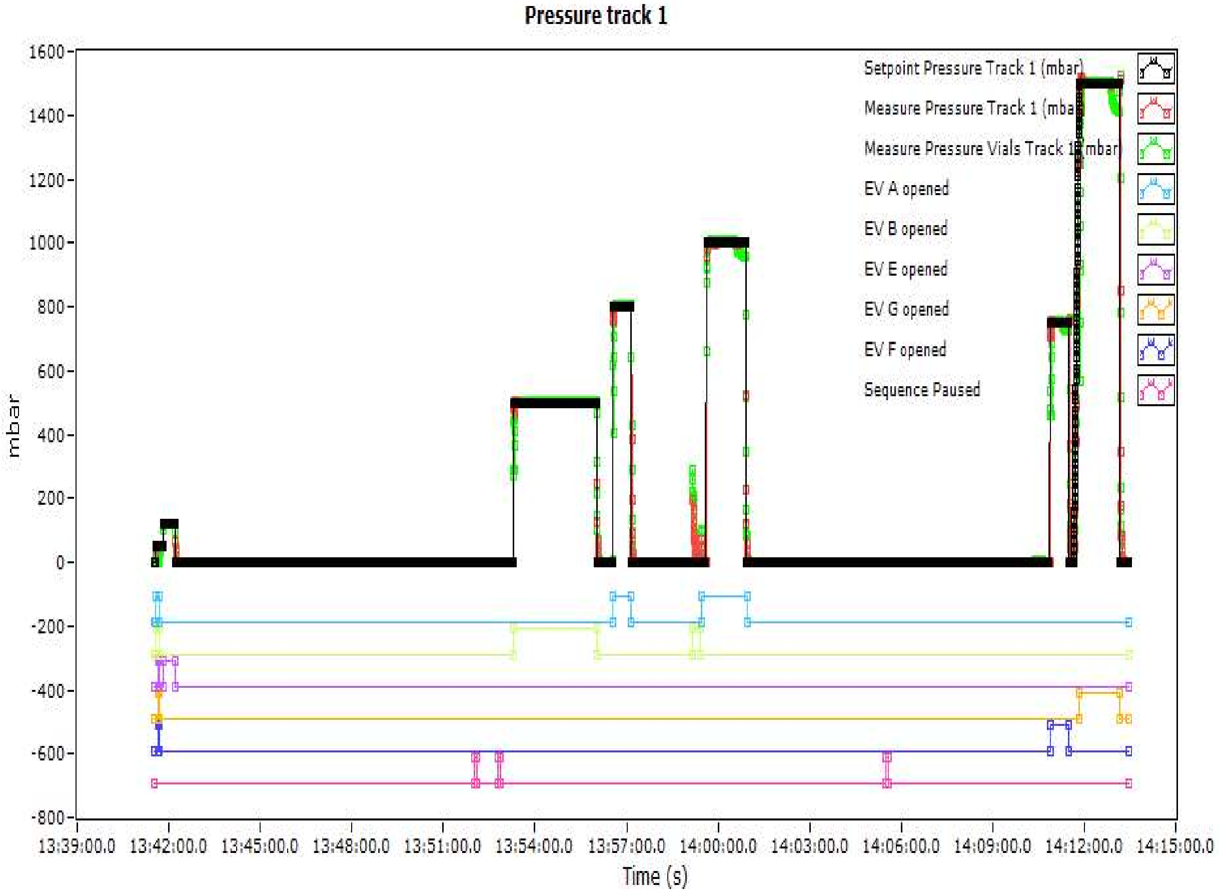

Pressure track 1

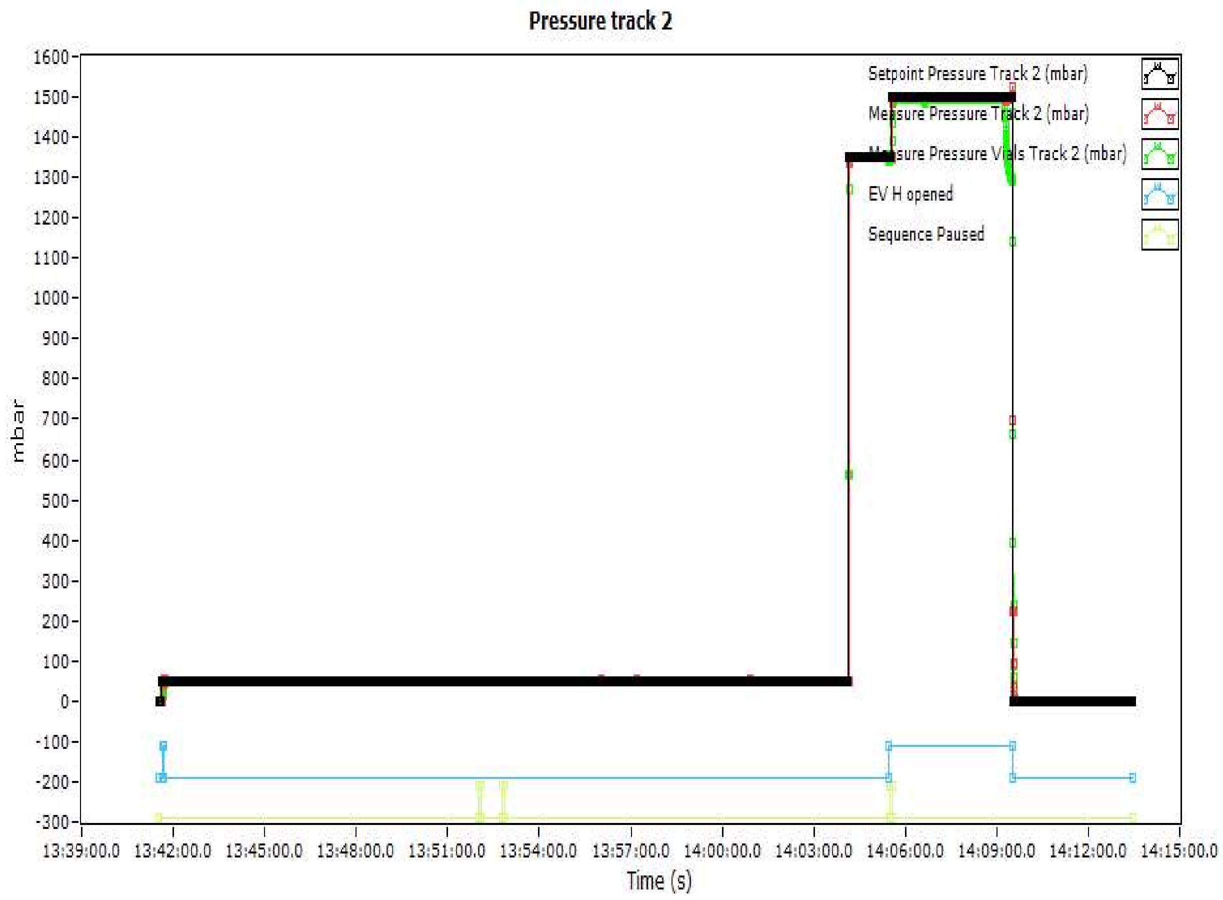

Pressure track 2

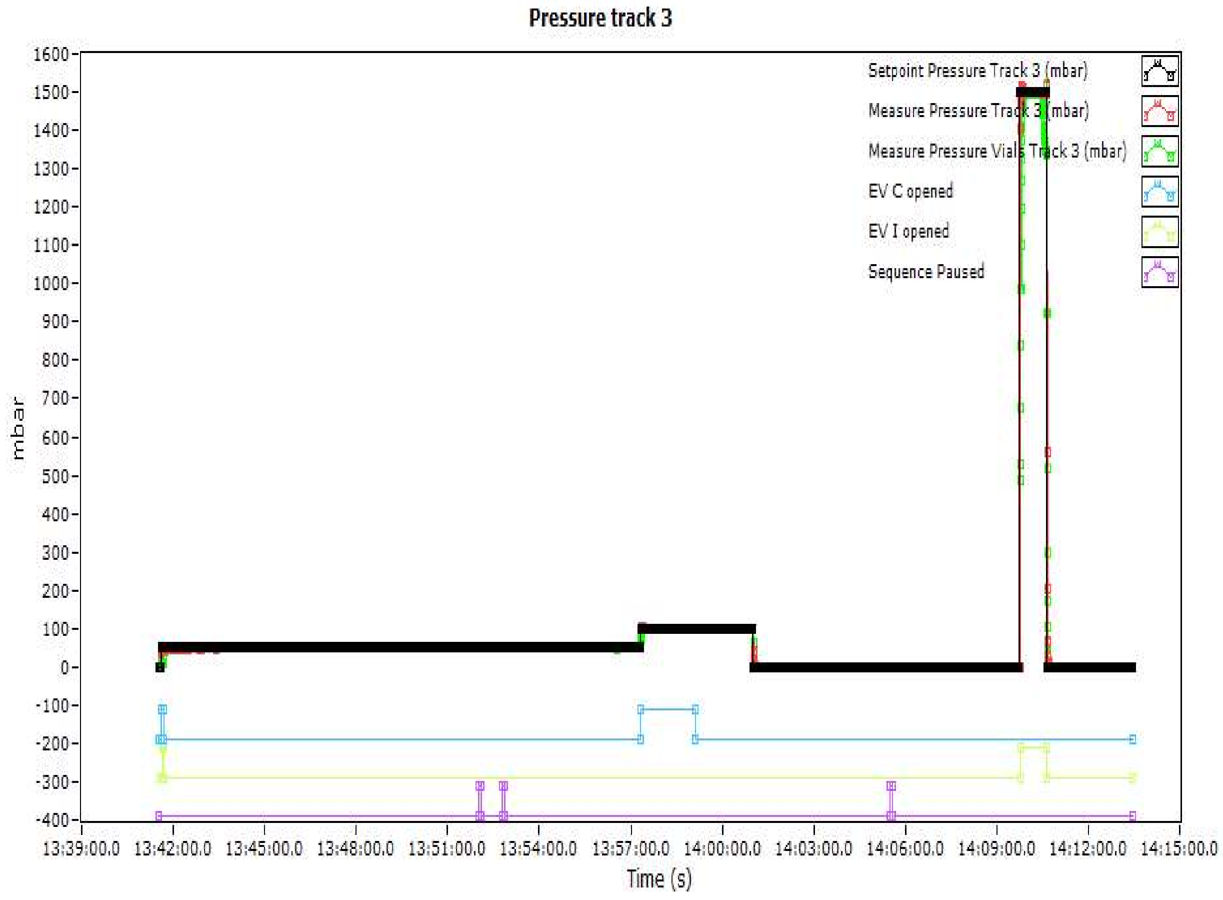

Pressure track 3

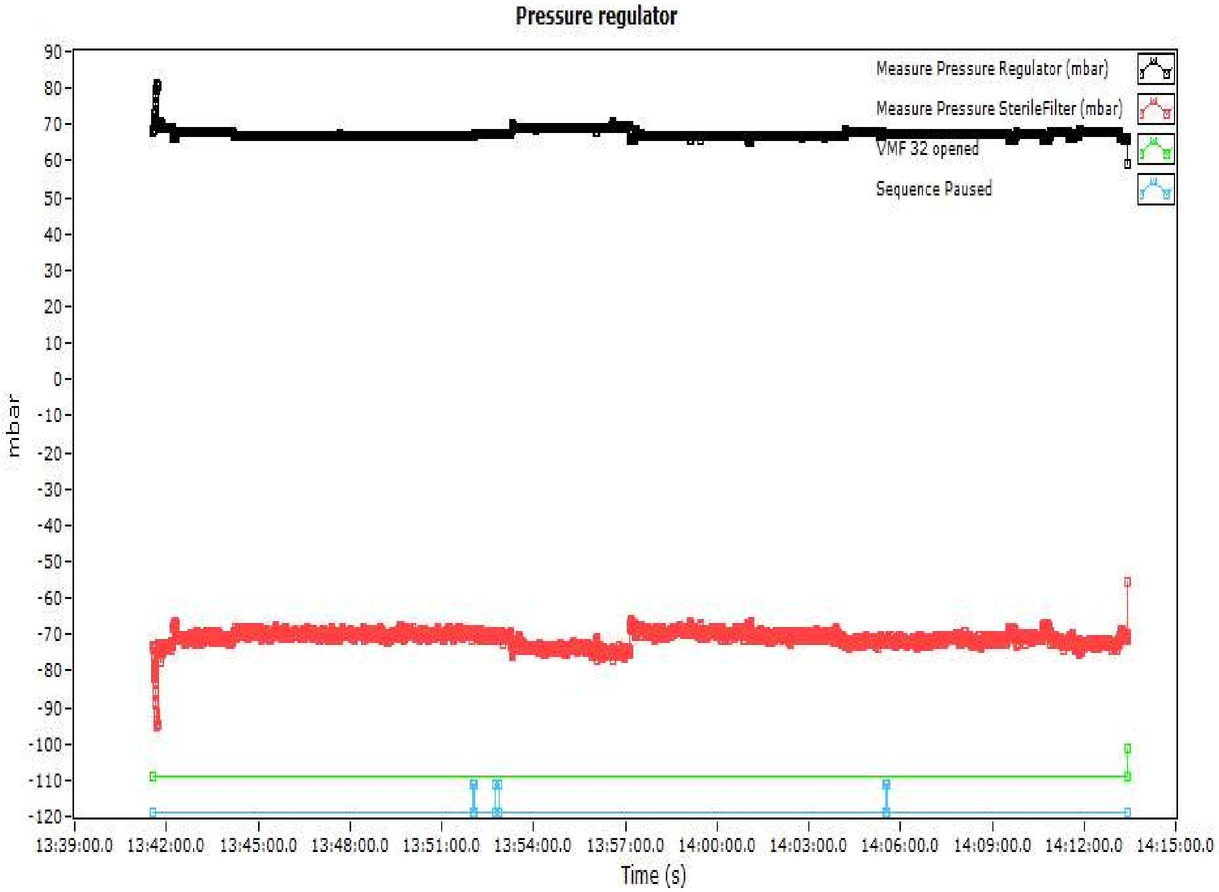

Pressure regulator

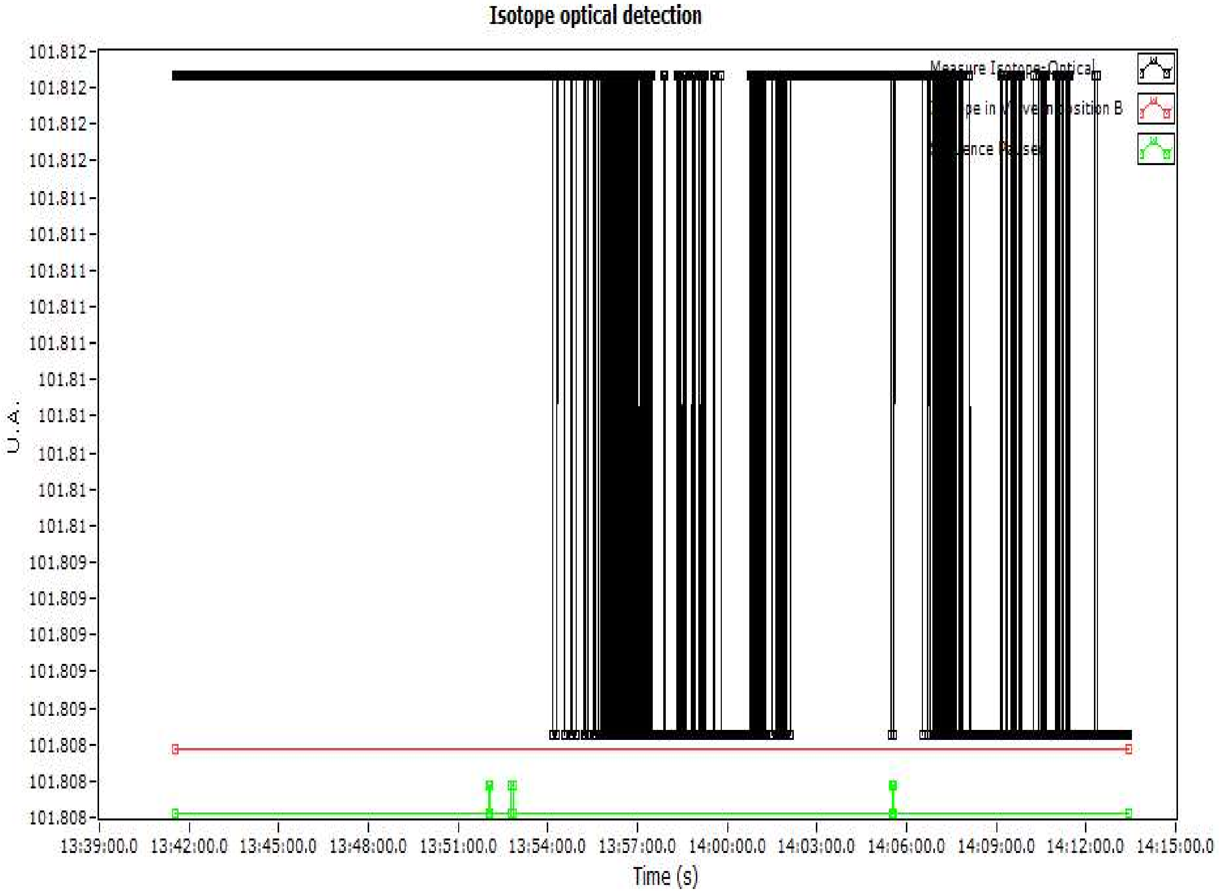

Isotope optical detection

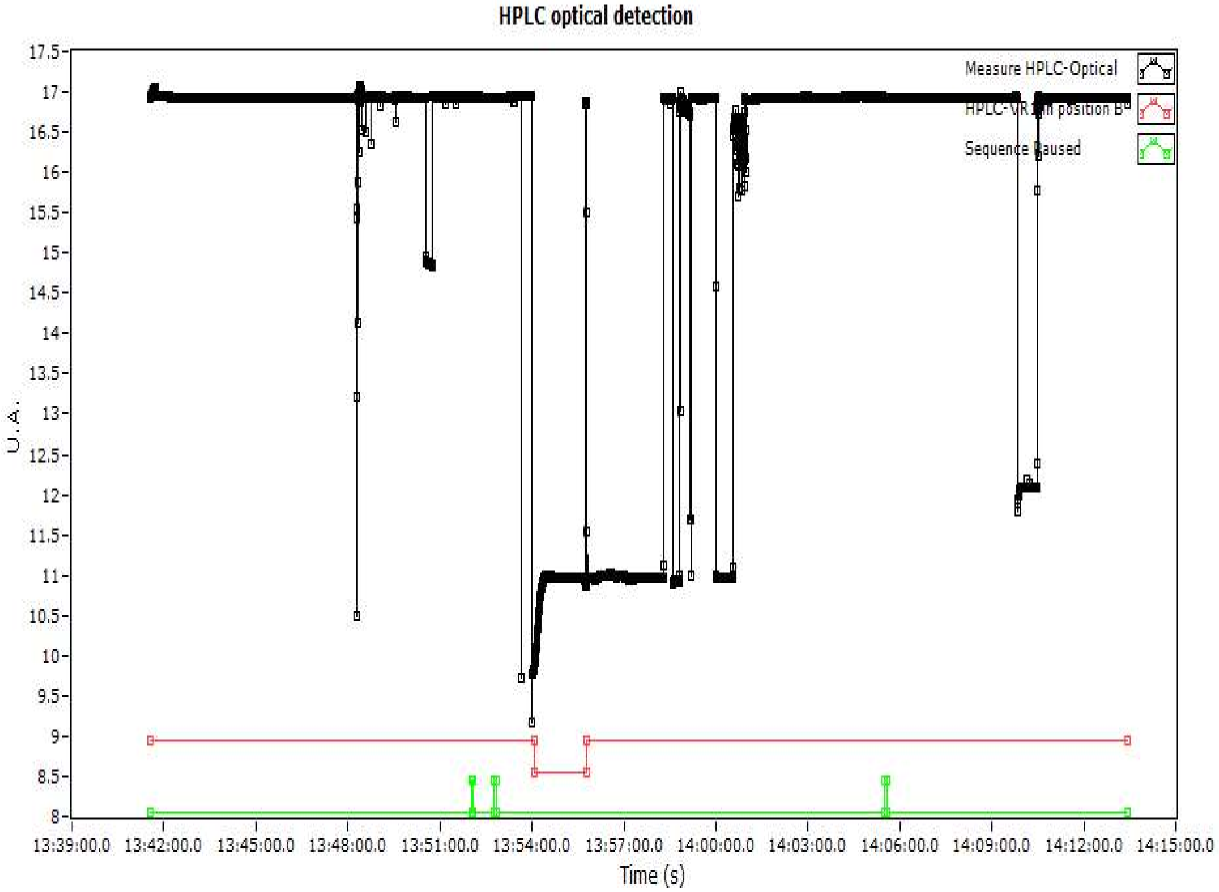

HPLC optical detection

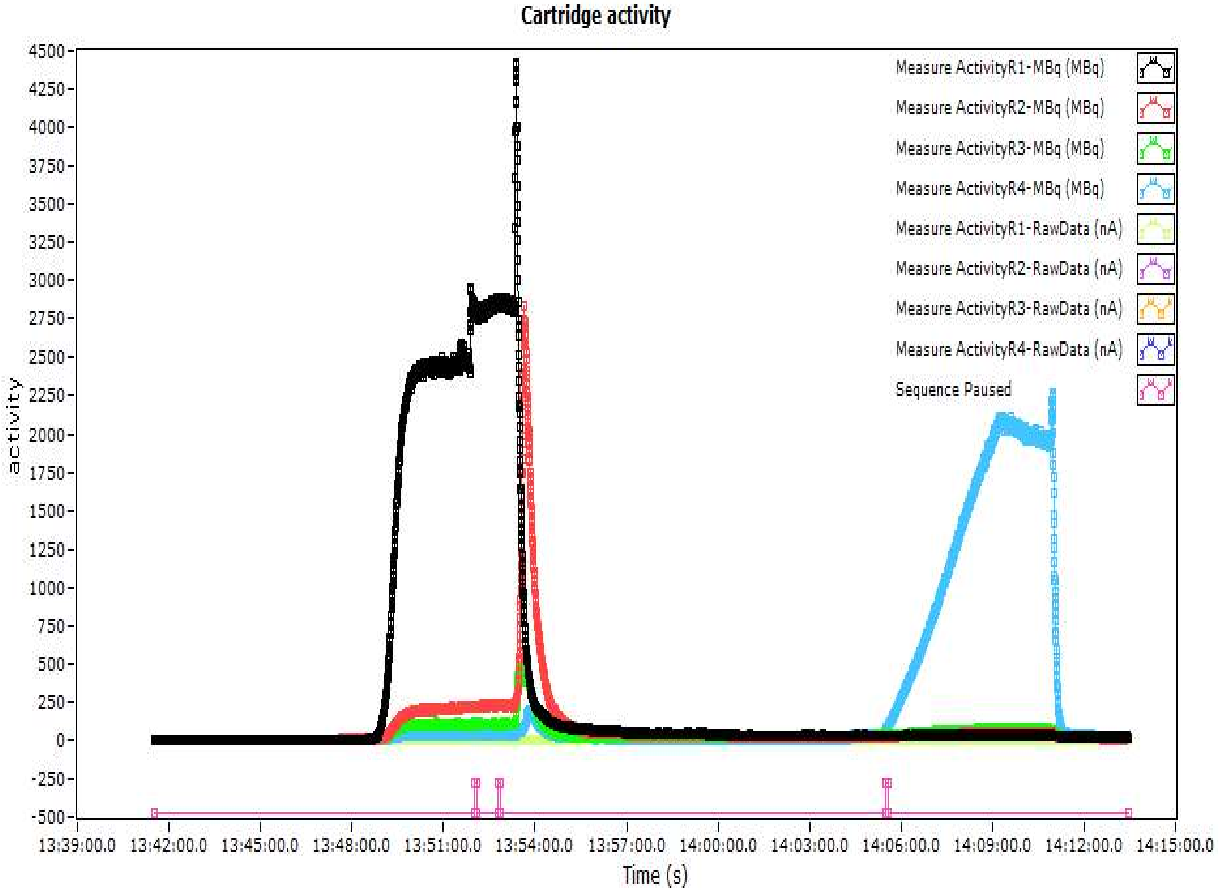

Cartridge activity

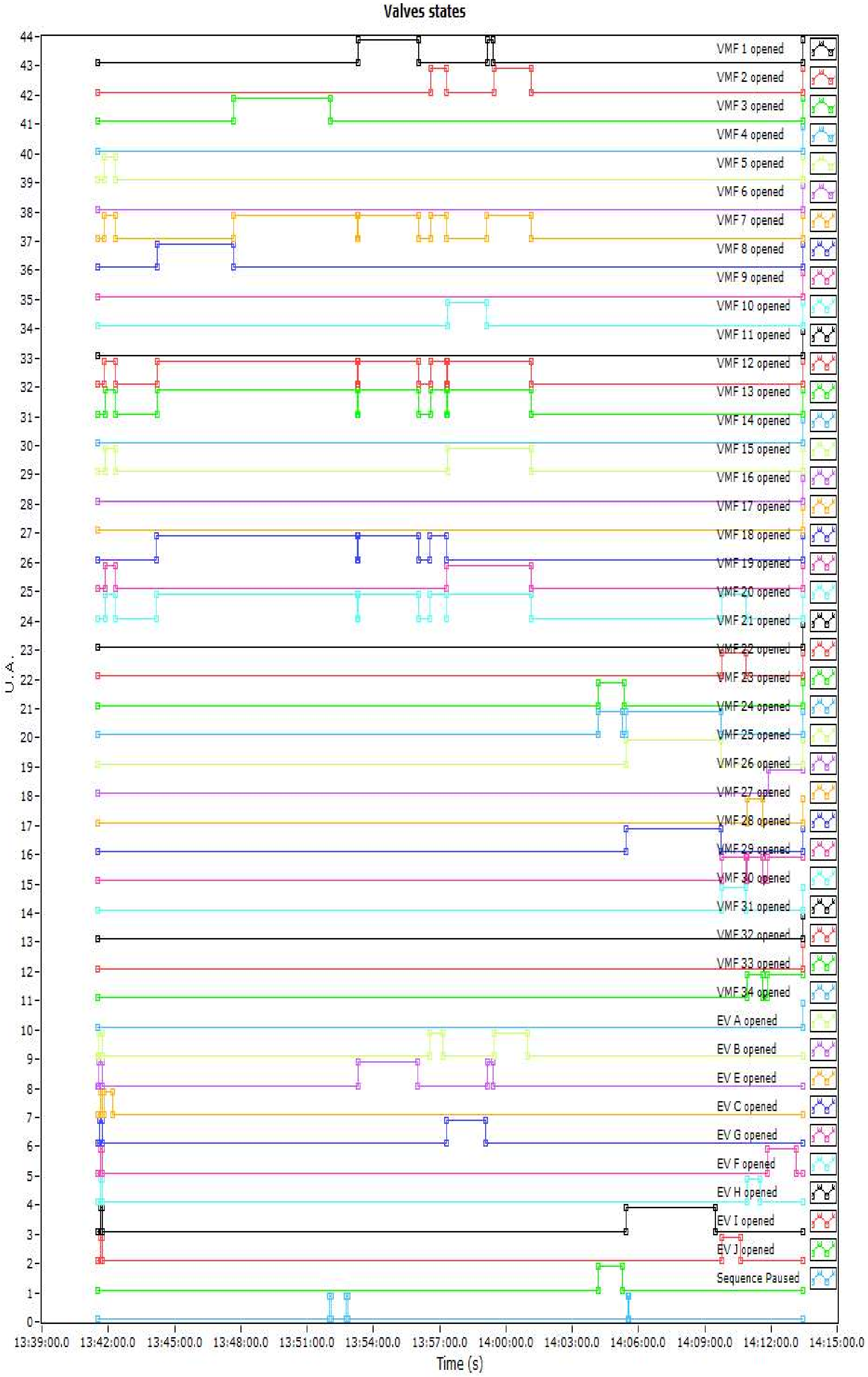
Valves states
U.A.
Time (s)
13:39:00.0
13:42:00.0
13:45:00.0
13:48:00.0
13:51:00.0
13:54:00.0
13:57:00.0
14:00:00.0
14:03:00.0
14:06:00.0
14:09:00.0
14:12:00.0
14:15:00.0
VMF 1 opened
VMF 2 opened
VMF 3 opened
VMF 4 opened
VMF 5 opened
VMF 6 opened
VMF 7 opened
VMF 8 opened
VMF 9 opened
VMF 10 opened
VMF 11 opened
VMF 12 opened
VMF 13 opened
VMF 14 opened
VMF 15 opened
VMF 16 opened
VMF 17 opened
VMF 18 opened
VMF 19 opened
VMF 20 opened
VMF 21 opened
VMF 22 opened
VMF 23 opened
VMF 24 opened
VMF 25 opened
VMF 26 opened
VMF 27 opened
VMF 28 opened
VMF 29 opened
VMF 30 opened
VMF 31 opened
VMF 32 opened
VMF 33 opened
VMF 34 opened
EV A opened
EV B opened
EV E opened
EV C opened
EV G opened
EV F opened
EV H opened
EV I opened
EV J opened
Sequence Paused

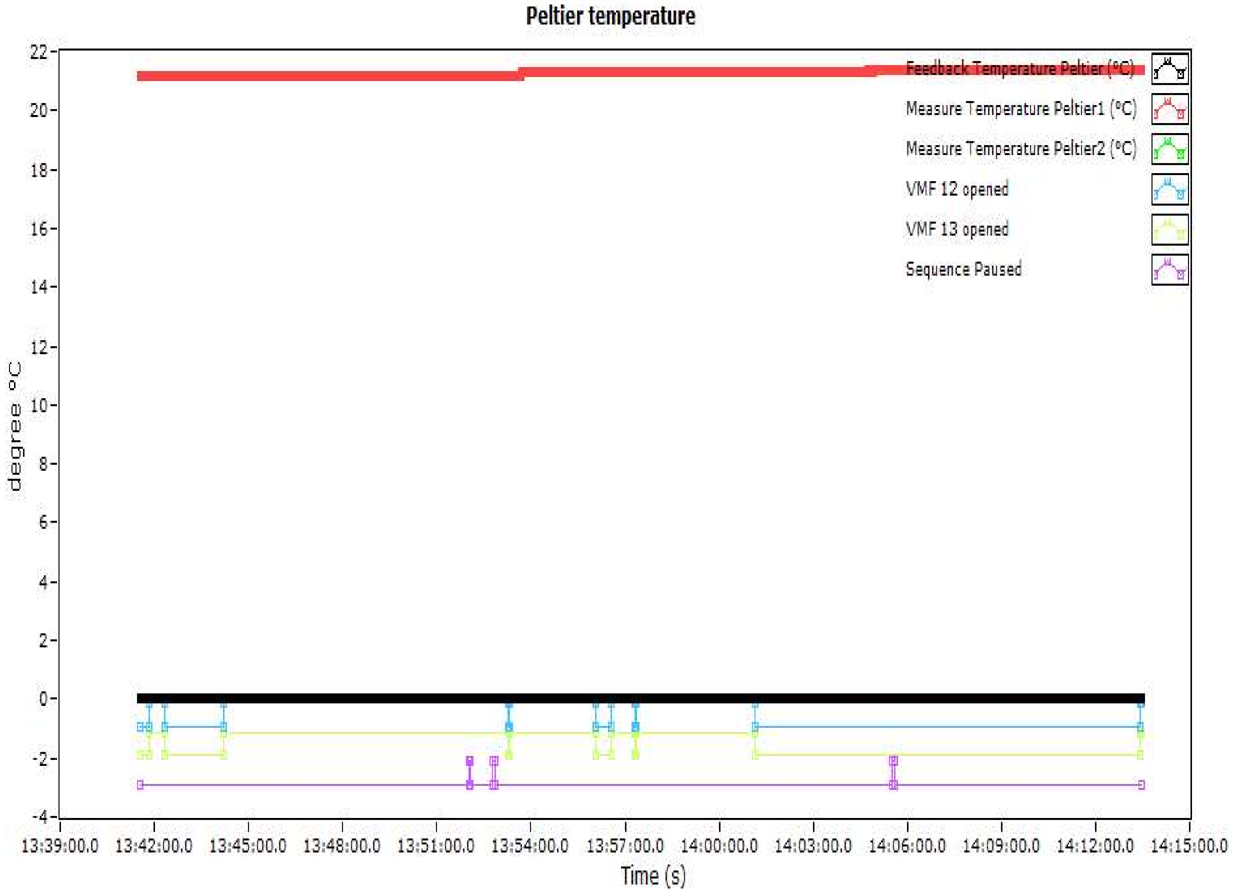

Peltier temperature

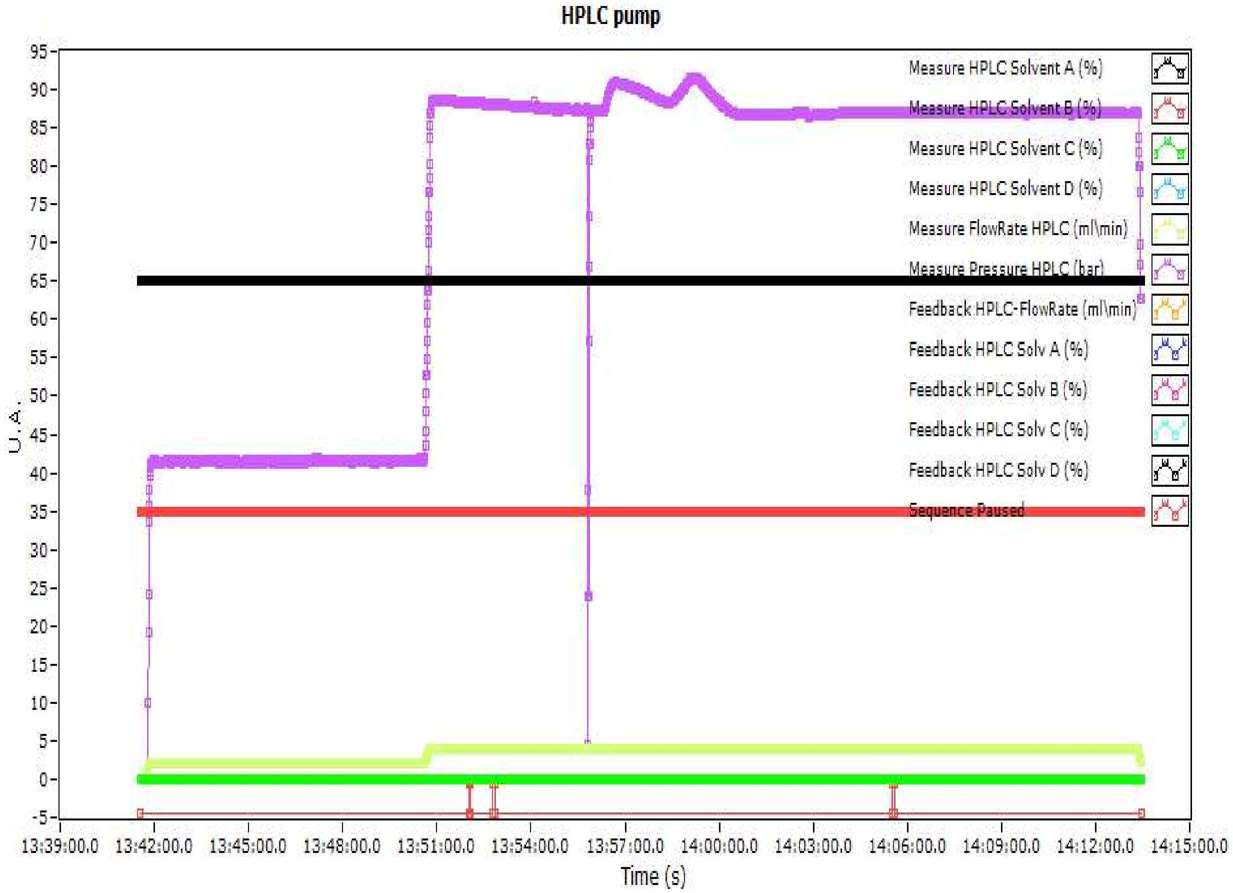

HPLC pump

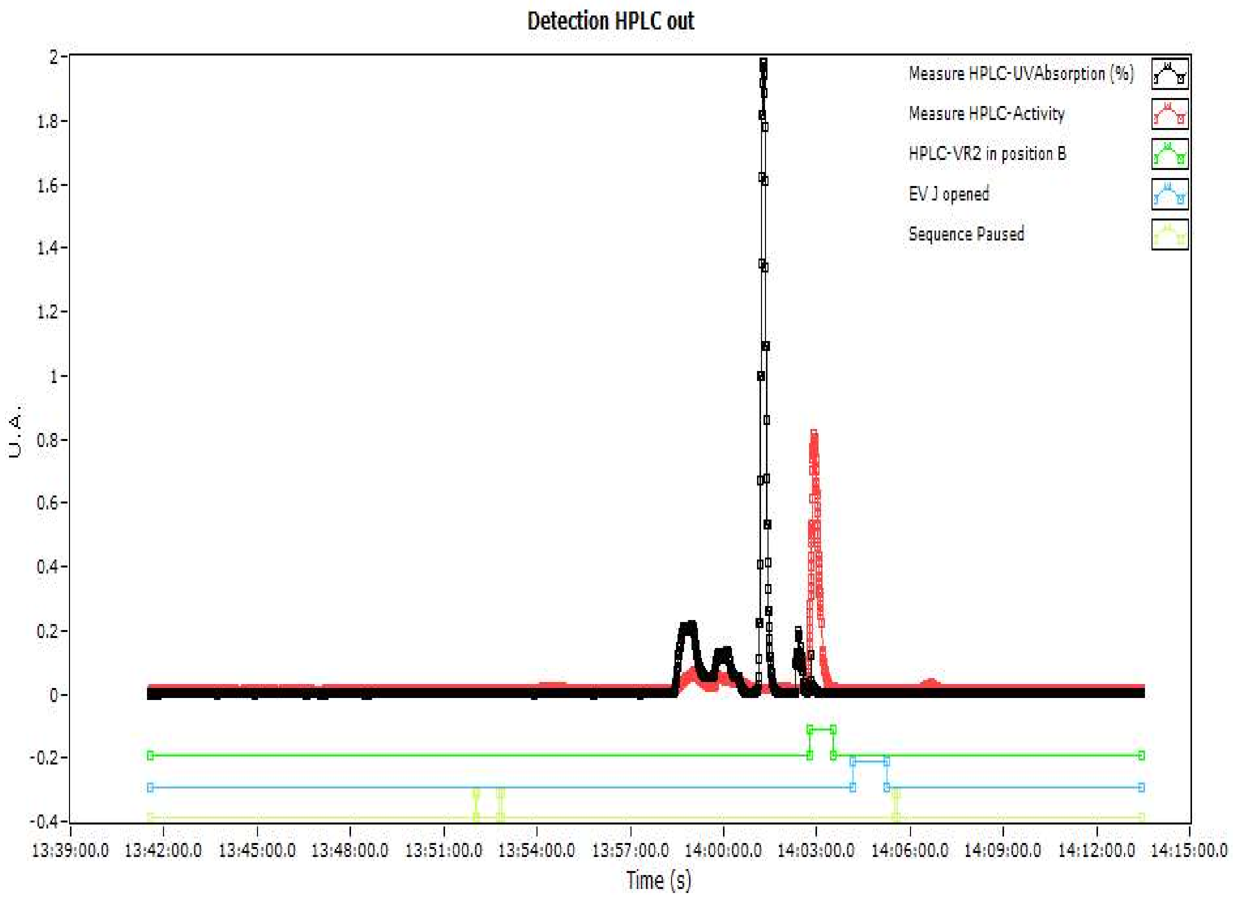

Detection HPLC out
